# Targeting NHE6 gene expression identifies lysosome and neurodevelopmental mechanisms in a haploid *in vitro* cell model

**DOI:** 10.1242/bio.059778

**Published:** 2023-11-29

**Authors:** Qing Wu, Li Ma, Lena Joesch-Cohen, Michael Schmidt, Ece D. Gamsiz Uzun, Eric M. Morrow

**Affiliations:** ^1^ Center for Translational Neuroscience, Carney Institute for Brain Science and Brown Institute for Translational Science, Brown University, Providence, RI 02912, USA; ^2^ Center for Computational Molecular Biology, Brown University, Center for Computational Molecular Biology, Providence, RI 02912, USA; ^3^ Department of Molecular Biology, Cell Biology and Biochemistry, Brown University, Providence, RI 02912, USA; ^4^ Department of Pathology and Laboratory Medicine, Alpert Medical School of Brown University, Providence, RI 02912, USA

**Keywords:** Christianson syndrome, NHE6, SLC9A6, RNA-seq, Lysosome

## Abstract

Christianson syndrome (CS) is an X-linked disorder resulting from loss-of-function (LoF) mutations in *SLC9A6* encoding the endosomal Na^+^/H^+^ exchanger 6 (NHE6). CS presents with developmental delay, seizures, intellectual disability, nonverbal status, postnatal microcephaly, and ataxia. To define transcriptome signatures of NHE6 LoF, we conducted in-depth RNA-sequencing (RNA-seq) analysis on a haploid NHE6 null cell model. CRIPSR/Cas9 genome editing introduced multiple LoF mutations into SLC9A6 in the near haploid human cell line Hap1. Isogenic, paired parental controls were also studied. NHE6 mutant cell lines were confirmed to have intra-endosomal over-acidification as was seen in other NHE6 null cells. RNA-seq analysis was performed by two widely used pipelines: HISAT2-StringTie-DEseq2 and STAR-HTseq-DEseq2. We identified 1056 differentially expressed genes in mutant NHE6 lines, including genes associated with neurodevelopment, synapse function, voltage-dependent calcium channels, and neuronal signaling. Weighted gene co-expression network analysis was then applied and identified a critical module enriched for genes governing lysosome function. By identifying significantly changed gene expression that is associated with lysosomal mechanisms in NHE6-null cells, our analyses suggest that loss of NHE6 function may converge on mechanisms implicated in lysosome-related neurologic disease. Further, this haploid cell model will serve as an important tool for translational science in CS.

## Teaser

**Summary:** We present an NHE6 null haploid cell line as a tool for basic and translational science relevant to Christianson syndrome. Transcriptome analysis implicates lysosome and neurodevelopmental mechanisms in cellular alterations.

## INTRODUCTION

Christianson syndrome (CS; OMIM 300243) is an X-linked neurodevelopmental disorder. Males with CS present with early developmental delay, nonverbal status, intellectual disability, epilepsy, progressive ataxia, postnatal microcephaly and hyperkinesis as core clinical features (Christianson et al., 1999; Pescosolido et al., 2014; EM and MF, 2018). Systematic sequence analysis of X-chromosome genes provides estimates of the frequency of CS at 1 in 16,000 to 1 in 100,000, making it among the more common X-linked intellectual disabilities (Tarpey et al., 2009). Currently, there are no treatments known that target the specific cellular causes of CS.

CS results from loss-of-function (LoF) mutations in the SLC9A6 gene which encodes the endosomal Na+/H+ exchanger 6 (NHE6) protein. To date, most of CS cases are caused by a spectrum of mutations, *de novo* or inherited, leading to a LoF in NHE6 (Pescosolido et al., 2014). NHE6 localizes primarily on the membrane of early, recycling, and late endosomes, and contributes to regulation of the luminal pH by moving H+ out of organelle in exchange for Na+ or K+ (Ouyang et al., 2013). NHE6 functions in trafficking intracellular cargo, as endosomes carry cargo either to plasma membrane or to lysosome for degradation.

Critical steps in a translational research pipeline often require a simple cellular model. Among the most prominent examples is the development of small molecule therapies using a simple cell-based *in vitro* assay for CFTR deficiency to develop therapeutics for cystic fibrosis (Langron et al., 2017; Phuan et al., 2018; Bacalhau et al., 2023). In addition, haploid cell models offer a powerful tool for genetic screening of phenotypic modifiers, such as suppressors and enhancers. Of course, a classic model in this regard is the application of yeast genetics, in particularly Saccharomyces cerevisiae, including application to neurological disease (Narayan et al., 2014). In mammalian cells, haploid cell models have recently emerged as a powerful tool for CRISPR/Cas9-based screens (Yin and Chen, 2017; Yilmaz et al., 2018; Bar et al., 2021; Brown et al., 2021; Llargues-Sistac et al., 2023).

In the current manuscript, we have established a haploid cell model with LoF mutations in the CS gene *SLC9A6*, encoding the endosomal Na+/H+ Exchanger 6 (NHE6). Here we present the validation of this model, and we have conducted in depth transcriptome studies. Gene expression profiling permits characterization of changes in gene expression in response to a perturbation which may serve as signature of gene mutation. High throughput RNA sequencing (RNA-seq) technologies help to provide an opportunity for an overview of the entire transcriptome. Using CRISPR/Cas9 genome-editing, LoF mutations were introduced into the SLC9A6 gene, inactivating the protein, in the near haploid Hap1 cell line. We have confirmed some of the predicted cellular phenotypes that may occur with loss of NHE6, such as over-acidification of intra-endosomal pH. The haploid cell model may easily permit genome-wide screens in a human cell.

In this study, even though our simple cell model represents a cancer cell line, we found that the expression of genes involved in neuronal processes, including axonogenesis, neuron differentiation, neuron projection morphogenesis, and neuron development, varied significantly in response to NHE6 mutations. Further and importantly, by constructing gene co-expression networks using the weighted gene-co-expression network analysis (WGCNA) approach, we identified dysregulated lysosome-related genes, significantly co-expressed in NHE6 null lines. There is some prior evidence of lysosome mechanisms implicated in CS pathogenesis (Stromme et al., 2011; Pescosolido et al., 2021; Fernandez et al., 2022). Therefore, in this study, we present a comprehensive and well-controlled analysis of the transcriptome architecture of NHE6 mutations in a new, human haploid NHE6 null cell line, which potentially provides insight into disease mechanisms in CS. In addition, this study provides a full analysis of a new cellular platform that may have utility in high-throughput drug screening, CRISPR-Cas9 screens or other translational experiments.

## RESULTS

### Establishment of NHE6 null near haploid human cell lines with paired, isogenic controls

To develop a relatively homogenous, isogenic *in vitro* cell line with NHE6 null mutations, LoF mutations were induced in SLC9A6 in Hap1 cells using genome-editing commercially by Horizon Discovery (Vienna, Austria). Hap1 is a near haploid human cell line derived from the male, chronic myelogenous leukemia (CML) cell line KBM-7 (Carette et al., 2011). We reasoned that the haploid nature of this line would potentially reduce the variability in cell line models, and thereby, enhance opportunity to see strong biological signals. In addition, future CRISPR/Cas9-based screens will be simplified by the haploid nature of the cells. *SLC9A6* mutations were generated in Hap1 cell lines by Horizon Discovery (Vienna, Austria) using CRISPR/Cas9-based genome editing. Three distinct mutant lines were established with paired controls (Table 1). This design with multiple lines and paired controls will help contend with variability across lines and to build a robust model with reproducible effects. Sanger sequencing from genomic DNA were performed to confirm mutations as shown in Fig. S1A. The mutant lines are MUT1, a single base pair deletion in exon 2 leading to a frameshift and premature stop codon in predicted transmembrane domain (TM) 3 (c.351delG, p.Tyr118Met fs*9); MUT4, a four base pair deletion in exon 2 leading to a frameshift and premature stop codon in predicted transmembrane domain (TM) 3 (c.351_354delGTAT, p.Tyr118 Ala fs*8); and MUT6, a single base pair insertion in exon 2 leading to a frameshift and premature stop codon in predicted transmembrane domain (TM) 3 (c.351_352insG, p.Tyr118Val fs*8) (Table 1). HAP1 parental control lines are commercially available together with the mutant lines from Horizon Discovery which enhances availability of these lines. As the vast majority of mutations in CS reflect numerous, distinct premature stop codons early in open-reading frame that lead to loss of mRNA, likely due to nonsense-mediated mRNA decay (NMD) (Lizarraga et al., 2021), these mutations have strong genetic construct validity with patient mutations.

**Table 1.**
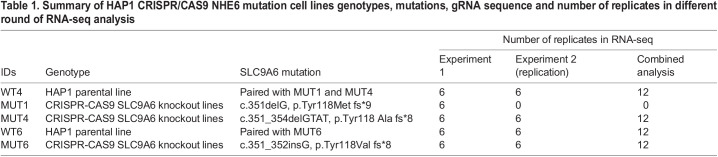
Summary of HAP1 CRISPR/CAS9 NHE6 mutation cell lines genotypes, mutations, gRNA sequence and number of replicates in different round of RNA-seq analysis

**Fig. 1.**
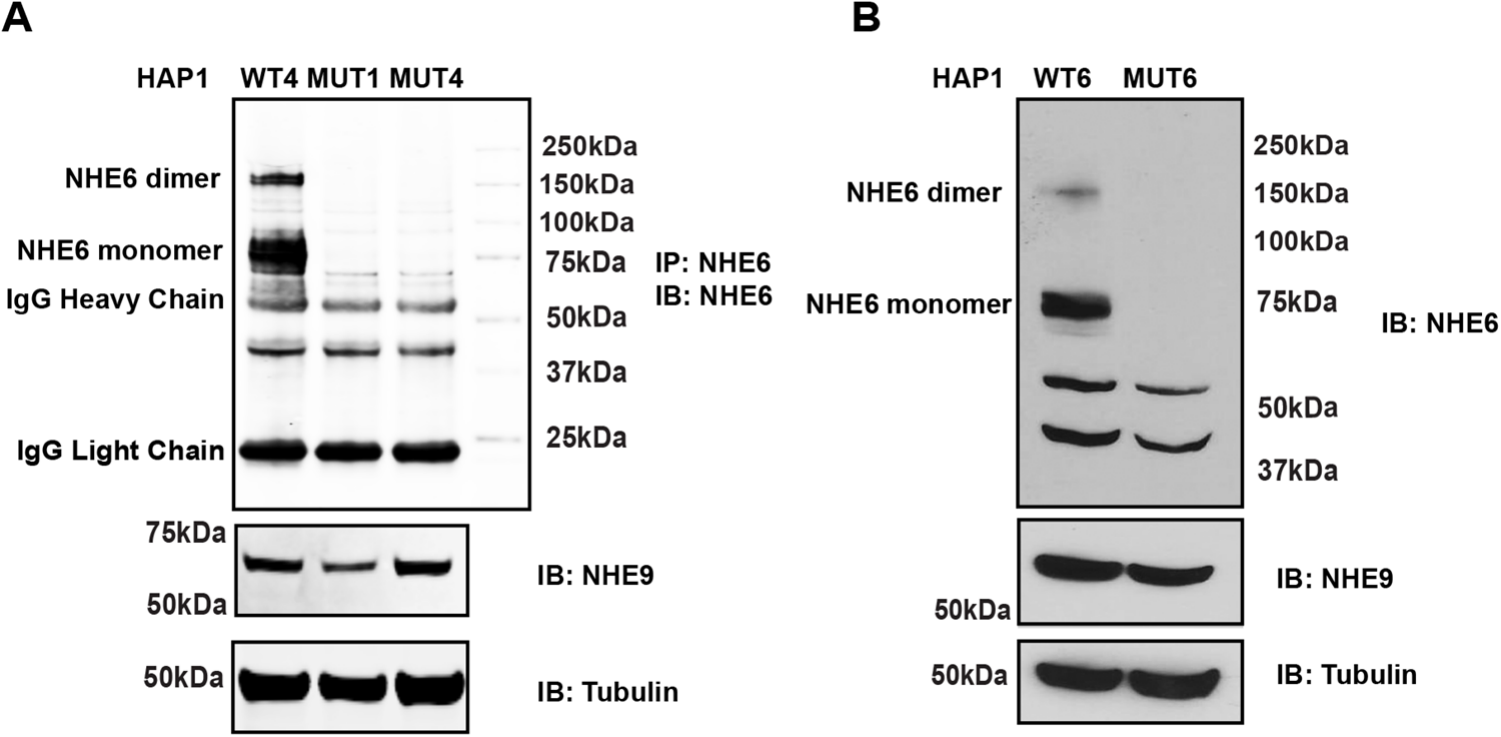
**Validated the mutation of NHE6 by immunoprecipitation of NHE6 and western blotting.** (A) Western blotting for NHE6 immunoprecipitates in the control HAP1 line (WT4), 1 bp deletion (MUT1) and 4 bp deletion (MUT4). (B) Western blotting for NHE6 in the control HAP1 line (WT6) and 1 bp insertion (MUT6).

We confirmed successful gene-targeting initially by loss of NHE6 protein. We have generated effective anti-NHE6 polyclonal antibodies to epitopes in the cytoplasmic tail of NHE6 (Ouyang et al., 2013). Immunoprecipitation of NHE6 followed by western blotting analysis reveals specific NHE6 protein products. Using these methods, we confirmed loss of NHE6 bands in all mutant lines (Fig. 1). Another closely related NHE family member, *SLC9A9*, coding NHE9, which is also localized in endosomes (Ohgaki et al., 2011; Donowitz et al., 2013), was detected to clearly show these knockouts were specific for NHE6 but not for NHE9, and notably, levels of NHE9 were not upregulated with NHE6 mutation (Fig. 1). While our antibody would not recognize potential N-terminal protein fragments, we believe that these truncated proteins are unlikely to be detected.

### NHE6 mutant cell lines demonstrate endosome lumen over-acidification

**Fig. 2.**
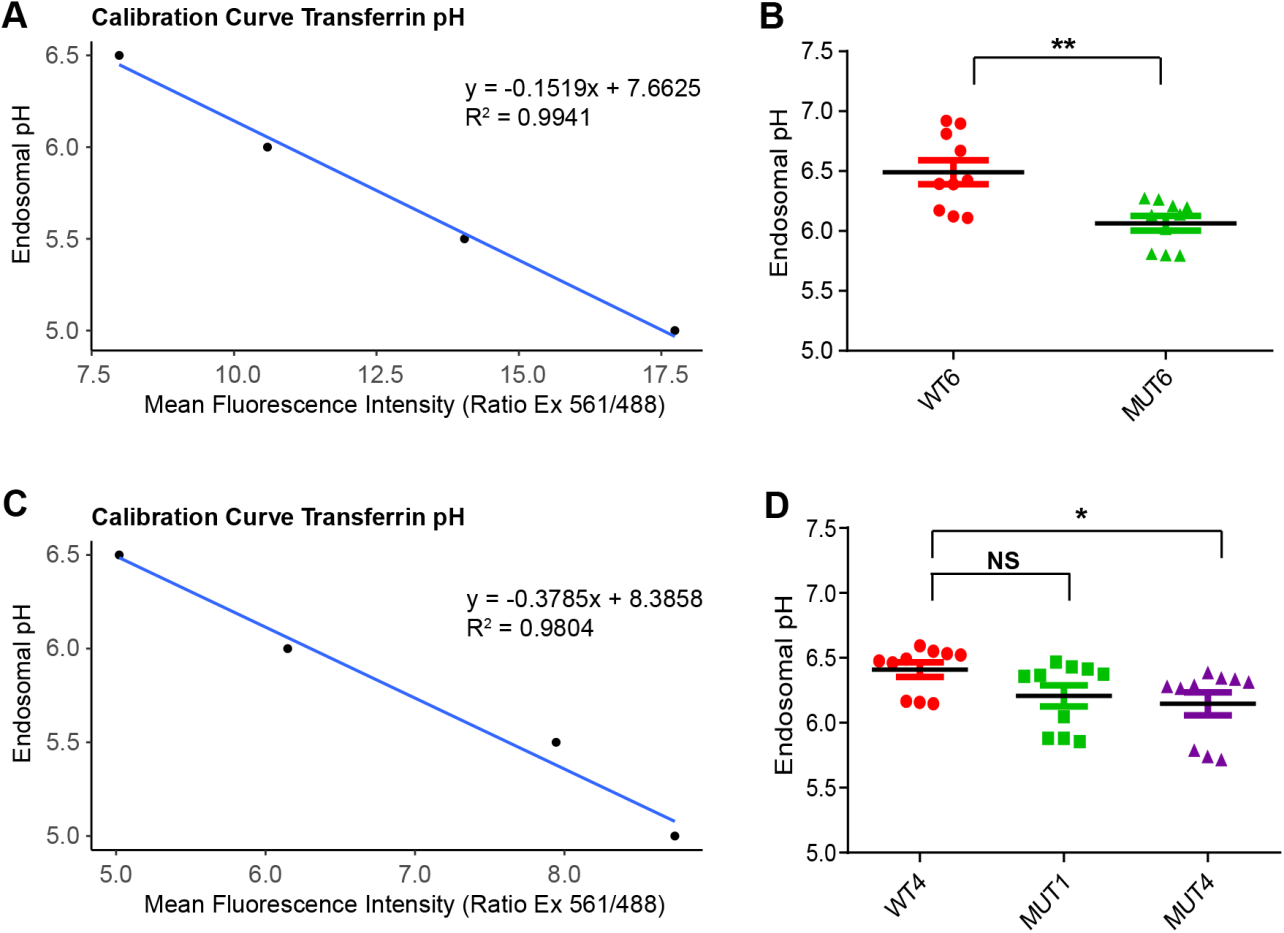
**Overacidification of endosomal pH observed in Hap1 mutant cells.** Endosomal pH with pH-sensitive (FITC) and pH-insensitive (Alexa 546) conjugates of transferrin were measured using flow cytometry. pH was calculated by measuring the mean intensity of Alexa-546 to mean intensity of FITC after generating pH standard curve. (A) Calibration standard curve and equation used to calculate the endosomal pH for MUT6 and control WT6. (B) Graph depicting endosomal pH for MUT6 and control WT6. *n*=3, unpaired *t*-test, *P*=0.0019. (C) Calibration standard curve and equation used to calculate the endosomal pH for MUT1, MUT4 and control WT4. (D) Graph depicting endosomal pH for MUT1, MUT4 and control WT4. *n*=3, unpaired *t*-test, *P*=0.0565 for WT4 versus MUT1, *P*=0.0211 for WT4 versus MUT4.

The Na+/H+ exchanger NHE6 is expressed on early, late and recycling endosomes, and provides a leak pathway for protons pumped in by the V-ATPase. We have previously shown in mouse primary neurons and human iPSC cells that loss of NHE6 is associated with over-acidification of the endosome lumen (Ouyang et al., 2013; Lizarraga et al., 2021). We then test if the endosomal lumen pH in Hap1 NHE6 mutant cell lines was also over-acidified. NHE6 WT and mutant cells were incubated with fluorescein-conjugated (pH sensitive) transferrin and Alexa-Fluor-546-conjugated (pH non-sensitive) transferrin for 10mins before sorting with Flow cytometry (FACS). Intra-endosomal pH was measured using mean fluorescence intensity ratio analyzed by FlowJo software (Sipe and Murphy, 1987; Xinhan et al., 2011; Ma et al., 2017). Standard calibration curves were prepared and equations for calculating endosomal pH were determined (Fig. 2A,B). The intra-endosomal pH was measured to be pH of 6.4907±0.0996 in WT6 and pH of 6.0639±0.0617 (*P=*0.0019) for MUT6 (Fig. 2C); and pH of 6.4094±0.0564 in WT4 as compared to pH of 6.2071±0.0816 in MUT1 (*P=*0.0565) and pH of 6.1460±0.0877 in MUT4 (*P=*0.0211) (Fig. 2D). Therefore, all three NHE6 mutant lines showed loss of NHE6 protein, and over-acidic endosomal lumen pH. Among all three mutant lines, MUT6 showed the strongest effect, MUT4, to a lesser extent, then MUT1 showed an effect on over-acidification of endosomal environment, which is based on average pH points change and statistically significant from three independent experiments (Table S1). These data further support the important function of NHE6 in maintaining intra-endosomal pH (Ouyang et al., 2013), also provide further validation of these cell lines as a model of NHE6 function and CS.

### Pathway analysis of differentially expressed genes (DEGs): NHE6 mutant lines reveal strong signature for neurodevelopmental mechanisms

**Fig. 3.**
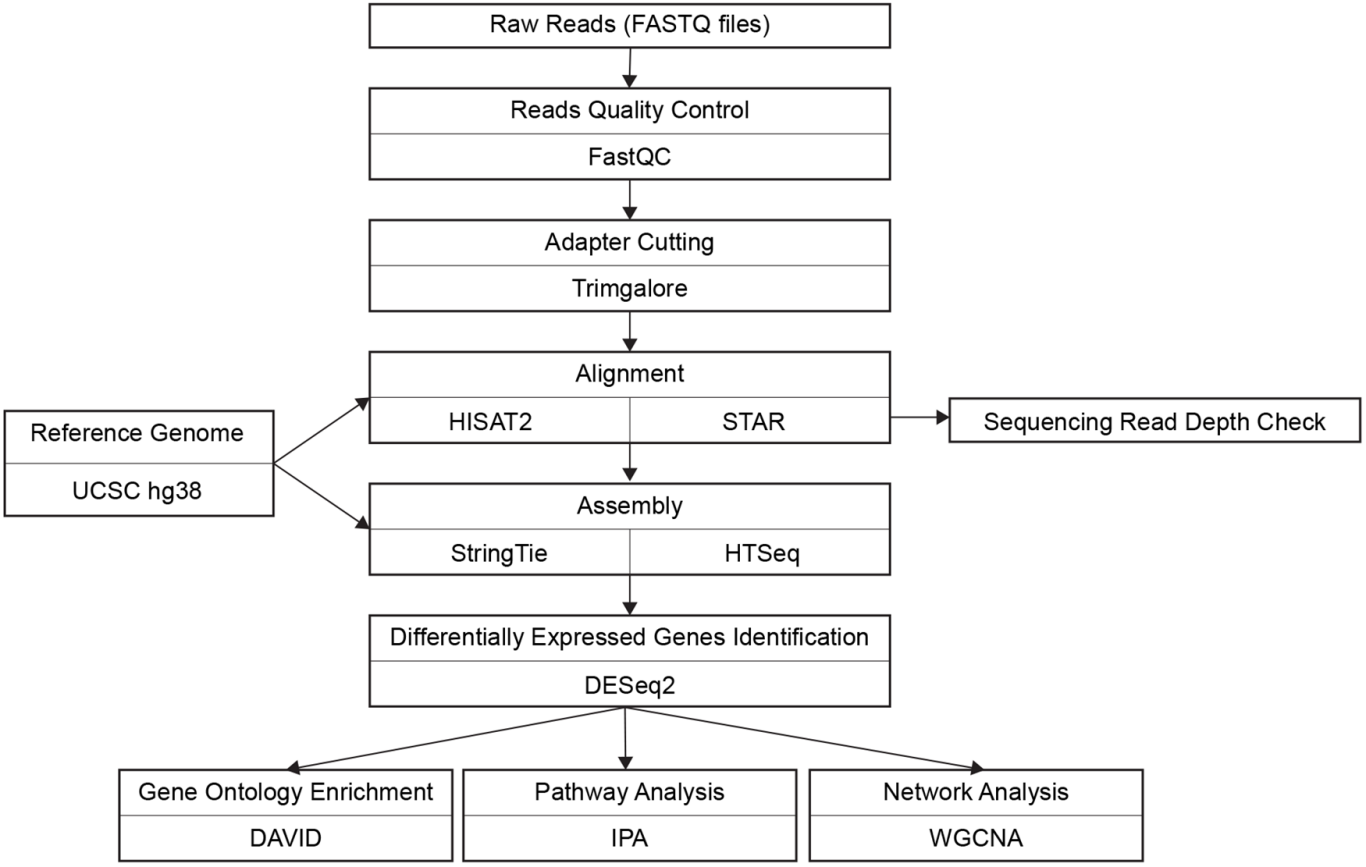
RNA-seq data analysis workflow including two separate pipelines.

In this study, we sought to identify genes that function with NHE6 in critical cellular mechanisms and/or to identify compensatory pathways resulting from NHE6 mutation. Therefore, we conducted an in-depth RNA-seq transcriptome analysis on NHE6 mutant cell lines to detect DEGs. Additionally, to enhance rigor further, we conducted a full biological replication of the RNA-sequencing, i.e. experiment 1 and replication experiment (Table 1). The samples were also analyzed in two separate pipelines, HISAT2-StringTie-DEseq2 and STAR-HTseq-DESeq2 (Fig. 3). Given the high level of replication across two experiments, we combined samples across two replications, i.e. the Combined Analysis, for the final analyses.

#### Experiment 1

In the first experiment, DEG analysis was conducted on MUT1, MUT4 and paired controls (WT4) with six biological replicates per line, as well as MUT6 and WT6 with six biological replicates per line. Alignment rates were 94.51% and 91.74% obtained from HISAT2 and STAR, respectively (Table S2). Mapping of sequence read depth on UCSC genome browser showed that SLC9A6 gene was drastically reduced across all three transcripts as described (Fig. S2).

**Fig. 4.**
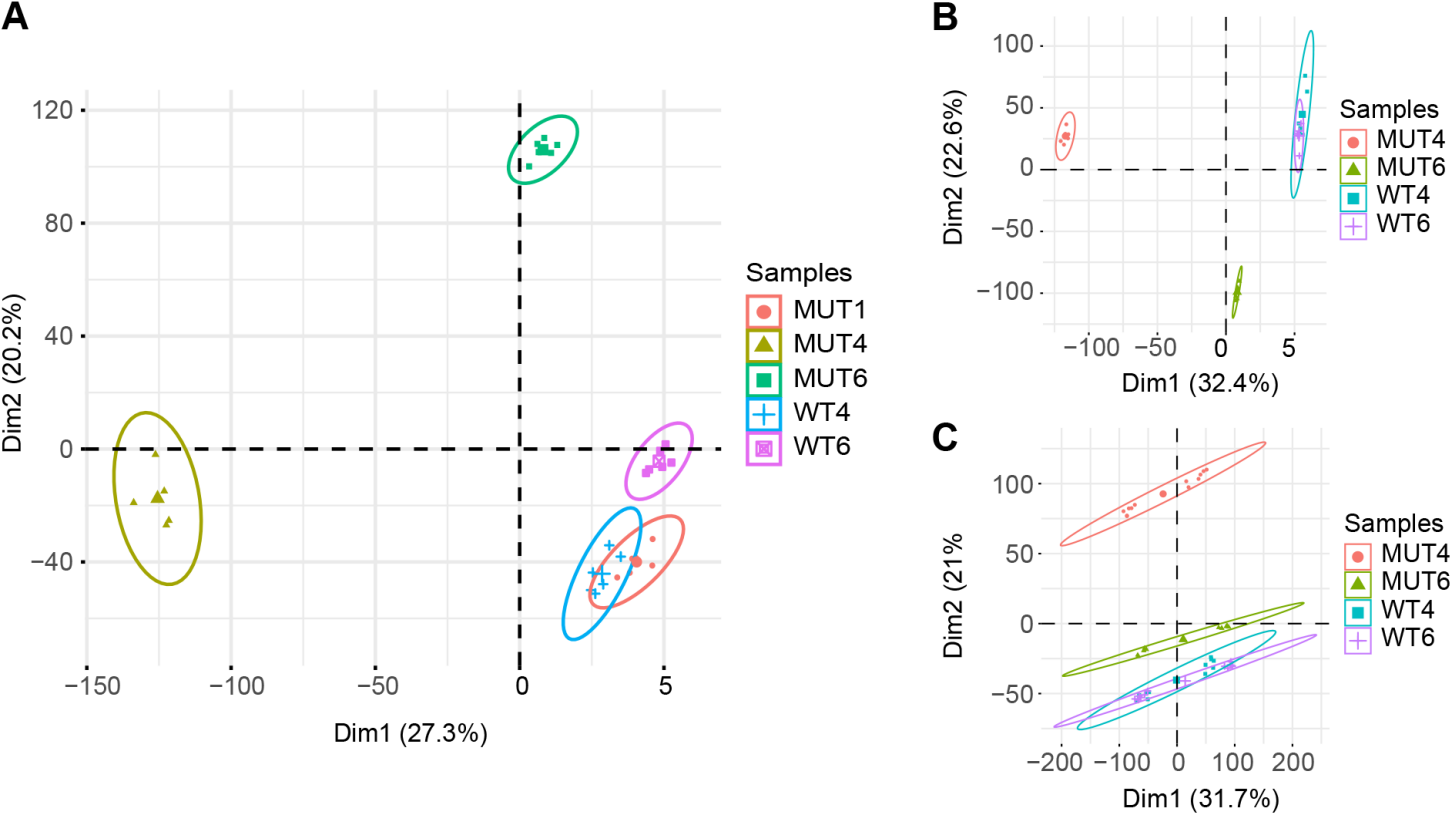
**PCA of (A) experiment 1, (B) replication experiment and (C) combined library experiment.** Each group has six replicates. X- and Y-axes represent first principal component and second principal component, respectively.

As part of the quality control, principal component analysis (PCA) was performed on a total of 14951 RNA-seq detected genes across 30 sequenced libraries of MUT1, MUT4, MUT6, WT4 and WT6 with six replicates. The first principal component clearly separates 4 bp deletion mutant group (MUT4) from the two wild-type groups (WT4, WT6), and the second principal component separates 1 bp insertion mutant group (MUT6) from the two wild-type groups (WT4, WT6) (Fig. 4A). However, the 1 bp insertion mutant group (MUT1) did not show separation with two control groups (WT4 and WT6). These results validate a distinct gene expression profile for two mutant lines (MUT4 and MUT6) from the control samples; however, it raises some concern that the third mutation line, MUT1, may have undergone additional changes *in vitro*, which normalized gene expression toward control.

603 DEGs, including 311 upregulated genes and 292 downregulated genes were identified using HISAT2 pipeline (Table S3). Using STAR pipeline, 586 DEGs were identified, including 272 upregulated genes and 314 downregulated genes (Table S4). (DEGs were defined as *P*.adj<0.01 and absolute log2 fold change>1.)

To confirm true differential gene expression in our pipeline, we conducted an independent gene expression assay, using a Nanostring methodology, on a large panel of select genes. We validated the RNA-seq DEGs using a custom-made panel of 44 genes and six housekeeping genes from Nanostring technologies. 44 genes include vATPase subunit, cathepsin and lysosome related genes (Table S5). The gene expression comparison between RNA-seq and Nanostring data are significantly correlated for mutant and wild-type lines (*P*<10^−7^) (Fig. S3). 12 out of 15 significantly DEGs (*P*.adj<0.05) identified in Nanostring were also identified as DEGs in RNA-seq and significantly correlated (*P*.adj<0.05). Therefore, this extensive independent validation of gene expression changes lends confidence to the RNA-seq DEG results.

#### Replication experiment

To augment the rigor of our studies, we set out to conduct a full replication of the RNA-seq experiments in MUT6 and MUT4 with paired controls. (MUT1 was dropped given the findings described above on the PCA.) The same analysis pipelines used in experiment 1 were run for this experiment. Alignment rates were 93.92% and 92.96% in HISAT and STAR, respectively (Table S2). PCA on a total of 15,203 RNA-seq detected genes across 24 sequenced libraries of MUT4, MUT6, WT4 and WT6 with six replicates validate a distinct gene expression profile for two mutant lines (MUT4 and MUT6) from the control samples (Fig. 4B). 1047 DEGs were identified in HISAT2 pipeline. Of these genes, 582 were upregulated, 465 were downregulated (Table S6). By comparison, using STAR pipeline, 1150 DEGs were identified. Of these genes, 624 were upregulated, 526 were downregulated (Table S7). (DEGs were defined as *P*.adj<0.01 and absolute log2 fold change>1.)

By sorting DEGs based on their *P* adjustment value, we found a concordance of DEG across experiment 1 and the replication experiment with a linear regression *P*-value less than 2.2e-16. Approximately 65% of DEGs identified in experiment 1 overlapped with those identified in replication experiment when HISAT2-StringTie-DESeq2 pipeline was used (Fig. S4A,C). The number of overlapping DEGs in experiment 1 and replication experiment was ten times as many as the number of overlapping genes if two gene sets were chosen randomly. Approximately 60% of the DEGs identified in both experiments overlapped when the STAR-HTSeq-DESeq2 pipeline was used. (Fig. S4B,D). The number of overlapped DEGs was also ten times as many as the number of overlapped genes when gene sets were chosen randomly.

#### Pathway analysis on the combined dataset

Given the high level of concordance across replications, we embarked on pathway analysis on the combined dataset, excluding MUT1. For the combined dataset, there were six biological replicates per line across two distinct rounds of sequencing (MUT6 and MUT4 with paired controls, WT6 and WT4), thereby reflecting a total of 12 sequenced libraries per sample. PCA was performed on a total of 14,171 RNA-seq detected genes across 48 sequenced libraries of MUT4, MUT6, WT4 and WT6 and confirmed two groups of MUTs were separate with two groups of WTs (Fig. 4C). The same analysis pipelines used in experiment 1 and replication experiment were run for the combined dataset. In total, 1056 genes were identified as DEGs by both HISAT2 pipeline (1132 DEGs, Table S8) and STAR pipeline (1148 DEGs, Table S9). Among them, 586 genes are upregulated, and 470 genes are downregulated in both pipelines. To reduce the bias and false positive data, we used the DEGs in agreement across both pipelines in the following pathway analysis.

1056 DEGs were analyzed in the Database for Annotation, Visualization, and Integrated Discovery (DAVID) and Ingenuity Pathway Analysis (IPA). We found neuron related processes, such as neuron differentiation (fold enrichment =2.33, *P*.adj=4.45E-05), neuron development (fold enrichment=2.25, *P*.adj=0.007), neuron projection morphogenesis (fold enrichment=2.77, *P*.adj=0.002) and axonogenesis (fold enrichment =2.85, *P*.adj =0.003) among the top gene sets in DAVID analysis. Enrichment of cell-adhesion related pathways such as biological adhesion, cell adhesion was also found in the top gene sets in DAVID analysis. Plasma membrane and synapse were the highly enriched gene sets among cellular component Gene Ontology (GO) terms. Most of these DEGs were shown to be involved in channel activity as the enriched molecular function GO term (Fig. S5A, Table 2). Neuron differentiation was the top enriched GO term in upregulated DEGs (fold enrichment=2.64, *P*.adj=0.0012) (Fig. S5B, Table S10). Plasma membrane part and synapse are the top GO terms for downregulated genes. (Fig. S5C, Table S11). We also tested the enriched GO terms in detectable genes list, the result is similar to the enriched terms above (Tables S12, S13, S14). Thus, pathways associated with these 1056 DEGs suggest neuron developmental pathways changes due to LoF mutation of NHE6.

**Table 2.**
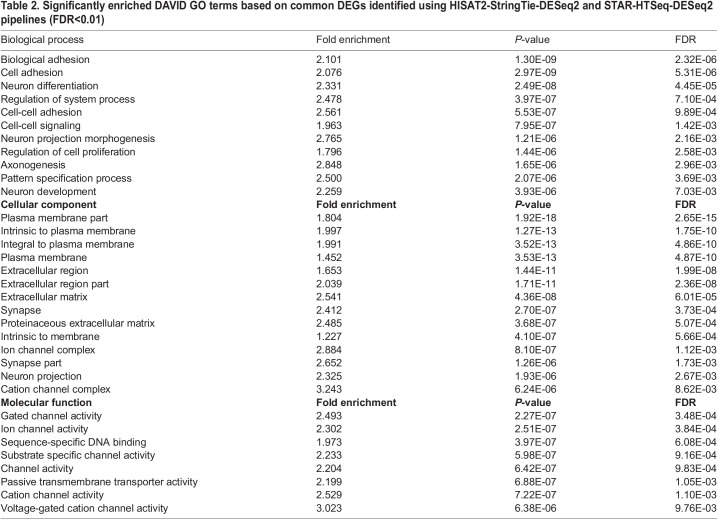
Significantly enriched DAVID GO terms based on common DEGs identified using HISAT2-StringTie-DESeq2 and STAR-HTSeq-DESeq2 pipelines (FDR<0.01)

We also analyzed 1056 significantly DEGs in IPA®. The top enriched pathway is axon guidance signaling pathway (*P*=3.1E-06). Axon guidance is a subfield of neural development, which is consistent with the findings from DAVID analysis. The following two overrepresented pathways are human embryonic stem cell pluripotency (*P*=4.15E-05) and GABA receptor signaling (*P*=7.42E-04), which is one of the main inhibitory neurotransmitters in a human's central nervous system. Several other overrepresented neurotransmitters related pathways are synaptic long-term depression (*P*=7.55E-04) and Reelin signaling in neurons (*P*=6.00E-03). (Table 3). Synaptogenesis signaling pathway is significantly enriched among downregulated DEGs (*P*=5.37E-05) and neurovascular coupling signaling pathway is significantly enriched among upregulated DEGs (*P*=3.47E-08) (Tables S15, S16).

**Table 3.**
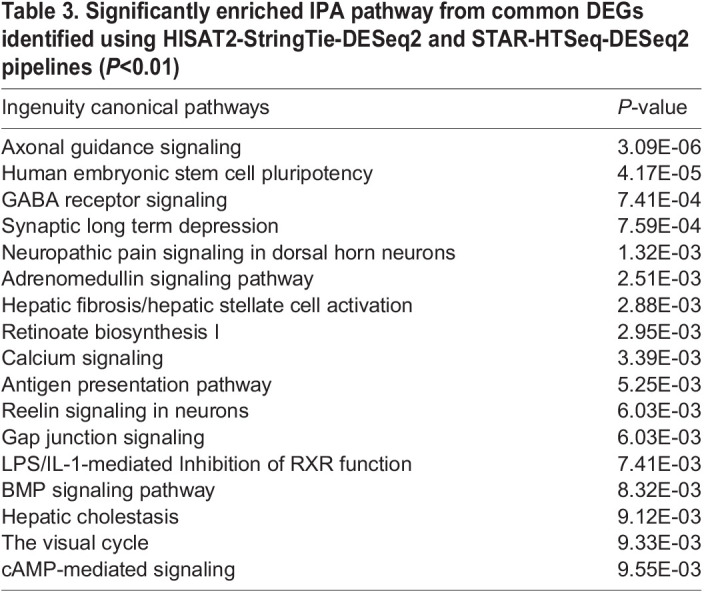
Significantly enriched IPA pathway from common DEGs identified using HISAT2-StringTie-DESeq2 and STAR-HTSeq-DESeq2 pipelines (*P*<0.01)

Therefore, even though the Hap1 line represents a cancerous cell, GO term enrichment analysis by DAVID, and canonical pathway enrichment analysis by IPA showed strong concordance for DEGs to be involved in mechanisms of neuronal development, particularly axonal and synaptic processes, secondary to mutation of NHE6, a gene mutated in the intellectual disability disorder, CS.

### WGCNA analysis: NHE6 mutant lines reveal strong changes in gene networks involving lysosome mechanisms

To study the co-expression relationships between genes at a system level, we performed WGCNA. WGCNA is a robust systems biology analysis method to obtain gene co-expression patterns based on a large dataset. It divided the whole gene set into modules based on the correlation between genes. The construction of a weighted gene correlation network was performed using the WGCNA R package (Zhang and Horvath, 2005; Langfelder and Horvath, 2008). 48 samples from combined dataset above, including MUT4, MUT6 and paired control WT4 and WT6 each with 12 replicates, were used in the network construction.

Hierarchical clustering was performed on all 48 samples to detect sample outliers. Although experiment 1 and replication experiment were not clustered together, MUTs and WTs were separated well in each experiment (Fig. S6). The result is in high concordance with the PCA plot. Genes with extremely low read counts, no variance in expression and below the noise threshold (normalized read count<10) across all 48 samples were excluded before the network construction. In total, 14,171 genes in 48 samples were used to create the weighted gene correlation network.

**Fig. 5.**
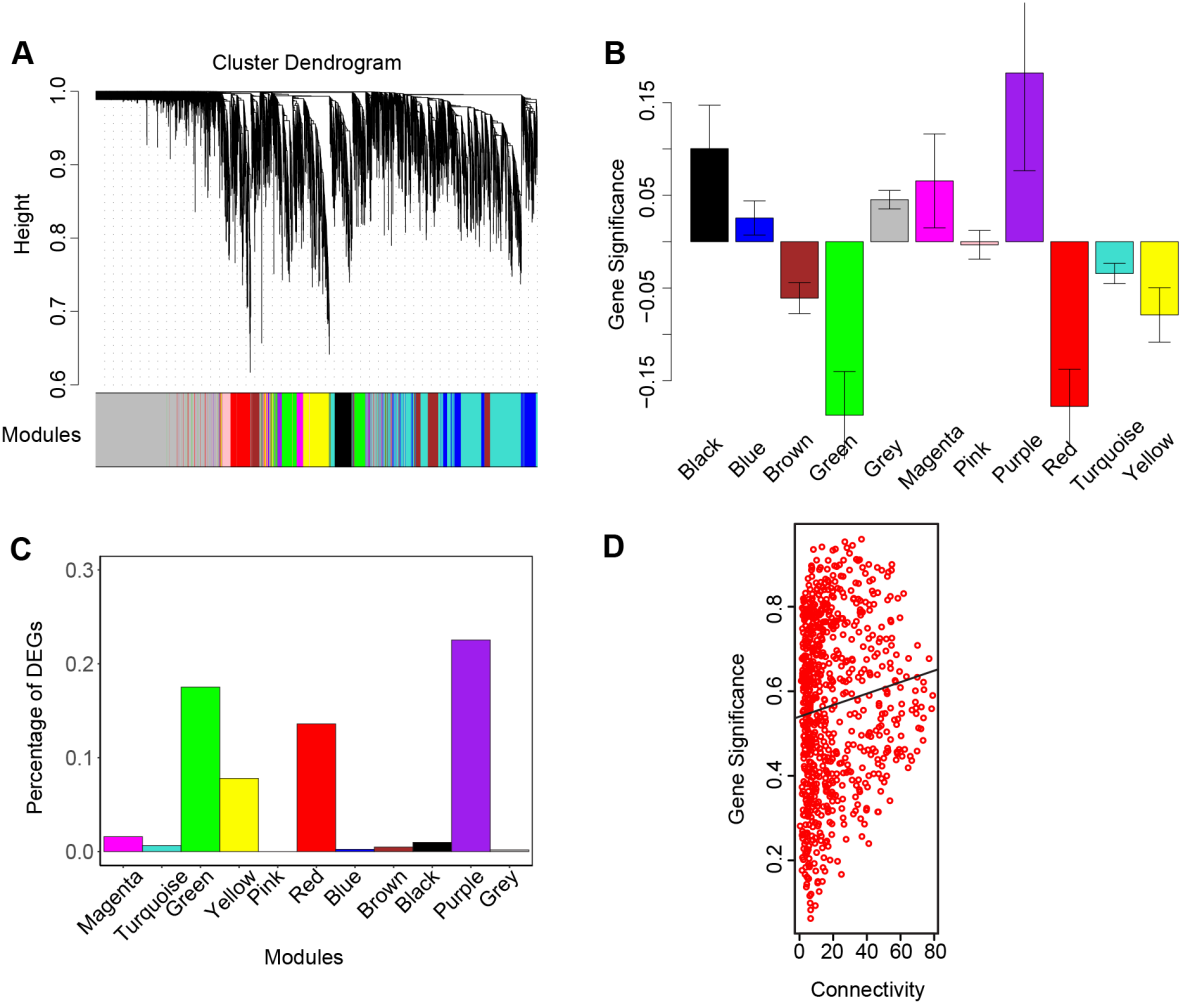
**Weighted gene correlation network analysis (WGCNA).** Each module is represented by its concordance color name. (A) Hierarchical clustering dendrogram showing the co-expression modules constructed by WGCNA. (B) The relative module significance (average gene significance) across modules. The Y axis is the relative module significance value. (C) Bar plot of percentage of DEGs across modules. The Y axis is the percentage of DEGs in each module. (D) Scatterplot of gene connectivity in red module. The X axis is the gene connectivity in red module and Y axis is the absolute gene significance value. Genes with higher connectivity shows a higher gene significance in the red module.

With WGCNA, the whole gene set was divided into 11 modules (Fig. 5A). SLC9A6 was clustered in purple module. Red module showed highly negative correlation with purple module with a correlation coefficient of −0.81 on a scale from −1 to 1, where 0 indicates low correlation and 1/-1 indicates high positive/negative correlation. Green, pink and magenta module with a correlation coefficient around 0.66 showed the highest positive correlation with purple module compared to others. Purple, red and green module among all modules showed the highest average gene significance value among all modules (Fig. 5B). 454 DEGs were selected after the noise threshold was applied. Only red, purple and green modules have DEGs percentage over 10% among all genes (Fig. 5C). Of these 454 DEGs, 107 DEGs were clustered in red module (13.6% of 787 genes in red module), 164 DEGs were in green module (17.52% of 936 genes in green module) and 55 DEGs were left to purple module (22.54% of 244 genes in purple module).

**Fig. 6.**
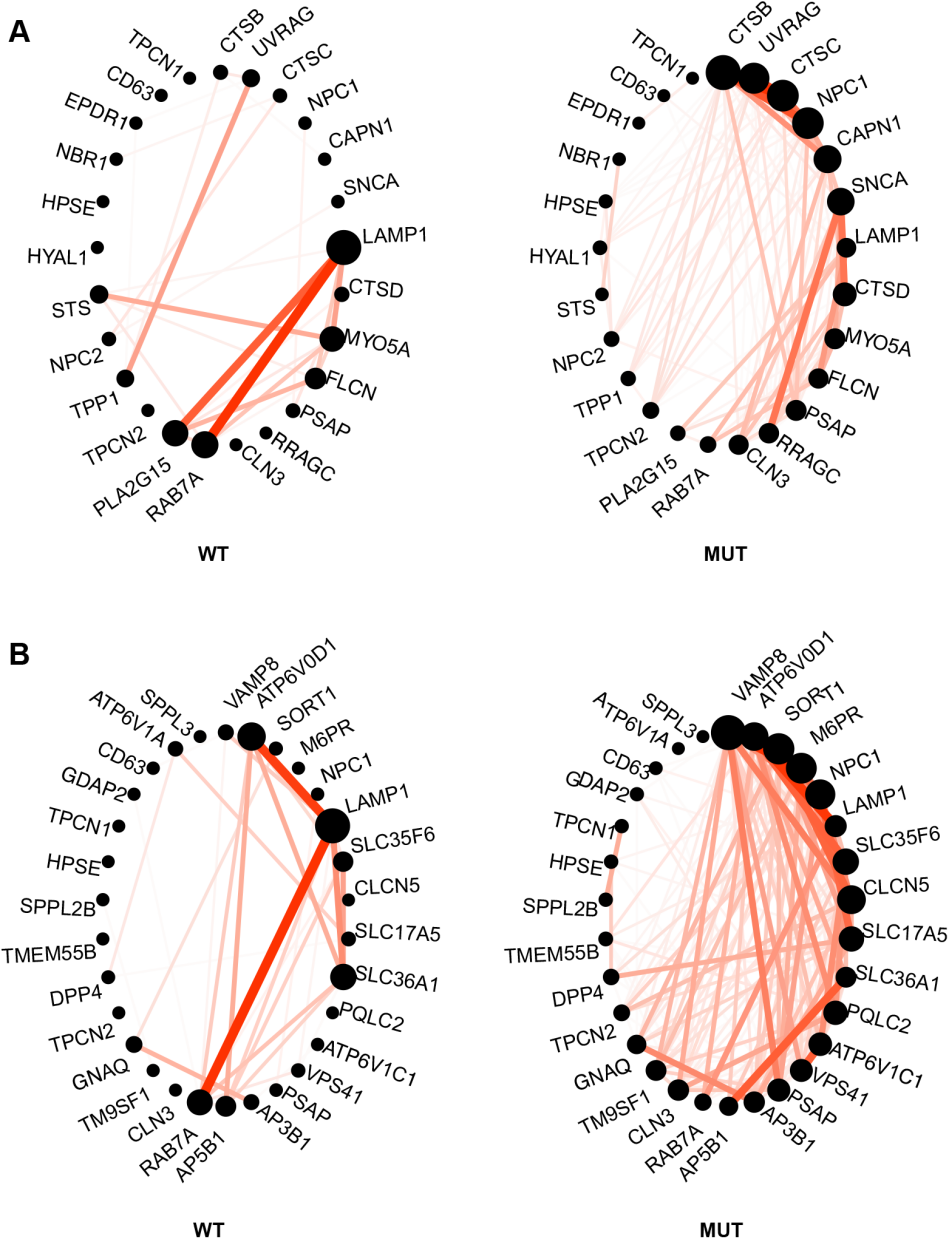
**Circle plot showed changes for enriched (A) lysosome genes and (B) lysosome membrane genes in mutant samples (MUT, right) compared to wild-type samples(WT, left).** In the circle plot, each dot represents a gene. The size of a dot represents a gene's connectivity within its module. A bigger gene dot represents a higher gene connectivity compared to a smaller gene dot. Red lines indicate the connection between two genes. A thicker and brighter color line between two genes indicates a higher correlation compared to a thin and saturated line. Both circle plot indicates big changes of lysosome and lysosome membrane genes in SLC9A6 mutant lines compared to that of wild type. The drawing of this circle plot was followed the circleplot.R provided by WGCNA.

GO enrichment analysis was run on genes in each module to identify the over-represented functional annotation of modules (Table S17). Three processes were identified at an *P*.adj significant level (*P*.adj<0.05) in red module (Table 4). Among them, two out of these three processes involve a cellular component at lysosome, indicating some changes in lysosome, especially in lysosome membrane. A circle plot was made using genes enriched in ‘lysosome’ (Fig. 6A) and ‘lysosome membrane’ (Fig. 6B). Compared to the wild-type circle, the overall connectivity among lysosome genes and lysosome membrane genes have increased in mutant circle. However, two major connections between LAMP1 and PLA2G15 and the connection between LAMP1 and RAB7A have decreased in mutant samples. Besides, two strong co-expression connections between 1) ATP6V0D1 and LAMP1 and 2) LAMP1 and RAB7A also decreased in mutant.

**Table 4.**
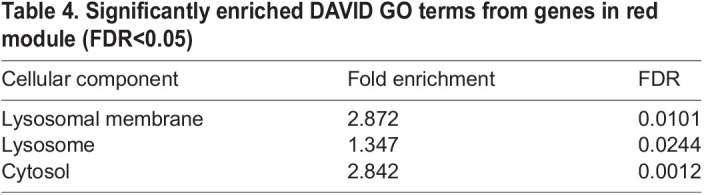
Significantly enriched DAVID GO terms from genes in red module (FDR<0.05)

## DISCUSSION

In this study, we provide in-depth molecular profiling using RNA-seq, and functional studies in a haploid cell line with LoF mutations in NHE6. This cell model may be used for large-scale screens related to cellular mechanisms or treatments in CS. CS is caused by a large number of distinct LoF mutations in the *NHE6* gene (Gilfillan et al., 2008; Pescosolido et al., 2014). While the mutations that we have established do not mimic specific nucleotide changes seen in CS, they do lead to LoF and thereby create strong genetic construct validity for the cellular effects seen in CS. Specifically, LoF mutations in CS appear to lead generally to loss of mRNA and protein by nonsense-mediated mRNA decay (NMD) (Lizarraga et al., 2021). In the current study, we see a complete absence of mRNA by RNA-sequencing (again reflecting likely NMD mechanisms), and loss-of-protein by western blotting. Since the antibody that we use to identify NHE6 in western blots recognizes the carboxy-terminal of the protein, we cannot formally rule out the possibility that a truncated protein is made in our cell models; however, given that we see a complete or near complete loss of mRNA, we believe that the possibility of translation of truncated protein is unlikely.

Several aspects of this new cell model are notable. First, this new NHE6-null cell model demonstrates a hallmark of NHE6 null cells, namely over-acidification of the early endosome compartment. NHE6 is localized to early and recycling endosomes and to lesser extent late endosomes (Ouyang et al., 2013). The acidification of endosomes is driven down by the vacuolar ATPase, and one model is that NHE6 opposes this acidification by permitting proton efflux. In a number of studies, loss of NHE6 function has been shown to cause relative acidification of the endosome (Ouyang et al., 2013; Lizarraga et al., 2021; Pescosolido et al., 2021). In the current study, across all three independent mutations of NHE6 in Hap1 cells, we observe over-acidification of the endosome. A second aspect of this new cell model is a robust resource is that we have established multiple independent mutations with paired control lines. When working with cell line models, one important consideration is the likelihood that the lines will compensate by subsequent mutations *in vitro*, even in early passages. To obviate this situation, good practice involves generation of multiple mutations and subclonal lines with isogenic paired controls. Of note here, while three distinct lines have been generated, one such line (the MUT1 line) appears to have potentially shown some reversion towards controls based on the PCA studies of the transcriptomes. MUT1 transcriptionally appeared closer to the controls as compared to mutants in our studies, which is why we excluded this line from our subsequent transcriptome analyses. Third, with regard to this resource, we have conducted an in-depth RNA-sequencing analysis, and these data have been deposited for wide sharing. While NHE6 is an endosomal protein, transcriptional changes reflect gene expression changes that result from the gene mutation and compensatory changes. This transcriptome dataset will now serve the research community to generate and test hypothesis related to NHE6 and endosome function in cells. While CS is most prominently a neurologic disorder, this simple cell line model may reveal important cellular signatures of NHE6 dysfunction, such as discovered here with regard to lysosome mechanisms. Interestingly, as described below, while our results clearly do reflect changes intrinsic to the context of a cancer cell line in culture, other changes pinpoint pathways reflecting neurodevelopmental processes. Finally, another potential strength of this model is the haploid nature of the cell line. It is possible that the transcriptional response to NHE6 mutation is simplified, and therefore, with a more prominent signal, by virtue of the fact that Hap1 cells are near haploid. At present, the extent to which this is true is difficult to know; however, the haploid feature of the line is important, and may facilitate subsequent CRISPR/Cas9 genome-wide enhancer or suppressor screens.

We have conducted two analyses of the transcriptome data: pathway analysis and co-expression network analysis by WGCNA. Importantly, we consider the changes in the gene expression to reflect molecular profiling, or cellular signatures of mutation of NHE6. We do not contend that NHE6 plays a direct functional role in gene transcription. From our analysis of DEGs, we identified a number of pathways involved in neuronal development and neurologic disease. Despite the fact that the cell context of the study is the Hap1 cell line, a cancer cell line, these results appear to indicate the NHE6-mediated transcriptional changes are prominently recognized as neuronal. We identified presynaptic proteins, such as neurexin (NRXN3), which is significantly increased (log2FC=5.72). DLG2, an important component of the postsynaptic density complex associated with postsynaptic membrane of excitatory synapse, is significantly decreased (Log2FC=−3.23). The expression of genes highly relevant to voltage-gated calcium channel activity, CACNA1B, CACNA1H, changed significantly. Calcium channel activity is associated with excitatory synapse function, secretion, neurotransmission physiological responses and highly associated with learning and memory (Nanou and Catterall, 2018). Mutations on CACNA1H was previously reported in epilepsy patients by influencing neuronal excitatory (Eckle et al., 2014). In neurons, EPHAs and EPHBs are reported associated with many neuronal processes including neuronal guidance events and synaptogenesis (Egea and Klein, 2007). The expression of EPHA3, EPHA7, EPHA8, and EPHB1 changed significantly in our CS cell model. Interestingly, the levels of RAB39B, which distributes on the secretary network at with ER/*cis*-Golgi interface and are colocalized with early endosome markers, decreased significantly in our mutant sample. Decreased function of RAB39B has been found to cause X-linked intellectual disability and early-onset Parkinson's disease through alpha-synuclein pathology (Wilson et al., 2014). We found the GO terms of neuron differentiation, neuron development, neuron projection, axonogenesis, synaptic function and gated channel activity was significantly enriched among DEGs. Canonical pathway by IPA showed the consistent result. Axon guidance signaling, GABA signaling, calcium signaling, synaptic long-term depression pathways are significantly enriched.

To explore the gene co-expression pattern, we created a network using WGCNA and identified modules that are highly co-expressed with NHE6. We found that genes in yellow module is associated with regulation of actin cytoskeleton, axon guidance events and growth cone. Pink module has genes associated with synapse formation and function. Brown module is significantly enriched with fatty acid biosynthesis process on endoplasmic reticulum and mitochondrion. Black module is identified associated with chromatin remodeling. Turquoise module is also highly associated with chromatin remodeling, chromatin modifications and negative regulation of transcription. Notably, there is growing evidence that chromatin regulation mechanisms crucially affect various stages of neural development, neuroplasticity, learning and memory (Ronan et al., 2013). Changes in translation process was found associated with autism (Santini et al., 2013). Genes involved with translation, including EIF4G1, EIF3G, SFPQ, TRA2B, are also identified in the turquoise module. Moreover, we found red module in our network contained genes that are responsible for lysosome functions, with a significant enrichment (*P*.adj<0.05) in ‘lysosome’ and ‘lysosome membrane’ GO terms. The level of lysosomal genes within red module are also significantly increased in the NHE6 mutant lines (absolute log2 FC<1, *P*.adj<0.01). Several vATPase genes are involved, including ATP6V0D1, ATP6V1A, ATP6V1C1. Genes for lysosomal proteases are increased, including CTSB, CTSC and CTSD, which is mutated in neuronal ceroid lipofuscinosis (NCL) type 10. In this network, we also noted upregulation of multiple lysosome disease genes, genes for related neurologic disease and other endolysosome genes: TPP1, the causation for NCL type 2; CLN3, the causation for NCL type 3; GDAP2, associated with spinocerebellar ataxia; CD63, RAB7A, LAMP1, M6PR, or PLA2G15. These associations with lysosome mechanisms and lysosome disease are in strong concordance with prior biological studies in CS mouse studies both *in vitro* (Pescosolido et al., 2021) and *in vivo* (Stromme et al., 2011). Therefore, these studies here, in a simple cell model, now provide valuable data supporting the idea that disruption of NHE6 leads to primary and fundamental defects in lysosome function, which is consistent with the argument from prior investigators who have argued that CS may be a lysosomal disorder or on a continuum with these disorders. While this prior data on lysosome dysfunction in neural tissue supports the relevance of the findings here, of course, other findings in this non-neural model will need to be investigated in neural models to explore further their relevance to brain and CS. By revealing the gene expression changes in a new NHE6-null cell model, our study provides a comprehensive resource for the study of NHE6 function. We also found transcriptional responses to NHE6 mutations implicate neurodevelopmental functions and lysosome-related mechanisms. We provide the data to public as a resource for the community of researchers studying NHE6, and for drug development.

Finally, this simple cell model of NHE6 mutation may serve as a valuable tool in high throughput screening for disease mechanism and therapeutic development studies. This study provides a full analysis of a new cellular platform which may have utility in high-throughput drug screening, CRISPR-Cas9 screens or other translational experiments. A translational science pipeline often requires a simple cellular model. A highly successful example is the development of small molecule therapies using a simple cell-based *in vitro* assay for CFTR deficiency to develop therapeutics for cystic fibrosis (Langron et al., 2017; Phuan et al., 2018; Bacalhau et al., 2023). In addition, haploid cell models offer a powerful tool for genetic screening of phenotypic modifiers, such as suppressors and enhancers. In mammalian cells, haploid cell models have emerged as a powerful tool for CRISPR/Cas9-based screens (Yin and Chen, 2017; Yilmaz et al., 2018; Bar et al., 2021; Brown et al., 2021; Llargues-Sistac et al., 2023). Here, we have provided an in-depth molecular profiling of an easy-to-use cell line that may serve in these important, high-throughput screens.

## MATERIALS AND METHODS

### Hap1 CRISPR/Cas9-edited *SLC9A6* knockout cell lines

Three Human Hap1 CRISPR/Cas9-edited *SLC9A6* knockout and two control lines were purchased from Horizon Discovery, Vienna, Austria. Cells were grown in Iscove's Modified Dulbecco's Medium (IMDM) supplemented with 10% fetal bovine serum (FBS) and 1% penicillin–streptomycin. Cell cultures were maintained at 37°C in a humidified atmosphere of 95% air and 5% CO_2_. Cells were passaged every 2-3 days and split at 70-75% confluency at the ratio of 1:10-1:15. Cell culture medium and reagents used for Hap1 cells were obtained from Thermo Fisher Scientific.

gRNA (5′- GTGGGCCTTGTGCTTCGGTA-3′) was used for generating three knockout lines: HZGHC004524c003 (with 4 bp deletion in exon2, c.351_354delGTAT, P.Tyr118 Ala fs*8, named as MUT4), HZGHC004524c011 (with 1 bp deletion in exon2, c.351delG, p.Tyr118Met fs*9, named as MUT1) and HZGHC004524c006 (with 1 bp insertion in exon2, c.351_352insG, p.Tyr118Val fs*8, named as MUT6). MUT1 and MUT4 share one control line, human Hap1 parental control c631, named as WT4, MUT6 has its own control line, human Hap1 parental control c631, named as WT6 (Table 1). We named two control lines differently according to different shipments.

### Sanger sequencing

Genomic DNA was extracted from Hap1 *SLC9A6* knockout cells (MUT1, MUT4, MUT6) and control cells (WT4 and WT6) using quick extract DNA extraction solution (Epicentre QE09050) and amplified by polymerase chain reaction (PCR). It was sequenced using Sanger methods. Primers for PCR amplification of exon 2 of *SLC9A6* followed our previous study (Pescosolido et al., 2014) (Exon2: forward 5′-atccatagttatgcgtgggg-3′ and reverse 5′-ctcctggatcattttgctgc-3′). Mutations were verified by the chromatogram using Chromas Lite software. The sequence was compared to *SLC9A6* sequence from UCSC/hg19.

### Western-blotting and immunoprecipitation

Human HAP1 *SLC9A6* knockout cells and two control cells were harvested. It was lysed in IP lysis buffer [50 mM Tris-HCl, pH 7.8, 137 mM NaCl, 1 mM NaF, 1 mM NaVO3, 1% Triton X-100, 0.2% Sarkosyl, 1 mM dithiothreitol (DTT), and 10% glycerol] supplemented with protease inhibitor cocktail and phosphatase inhibitor for 30 min on ice. Cell lysates were generated by centrifugation at 13, 200 rpm for 15 min at 4°C. Protein concentration was measured by BCA assay using the Pierce BCA Kit (Thermo Fisher Scientific 23225). For immunoprecipitation, 4 ug of custom-made rabbit anti-NHE6 antibody (C-terminal epitope: GDHELVIRGTRLVLPMDDSE, Covance 048) (Ouyang et al., 2013) were conjugated with 0.5 mg Dynabeads^®^ Protein G (Thermo Fisher Scientific) at room temperature (RT) for 2 h. After washing the beads, 500 µg of protein lysate was incubated with beads overnight at 4°C and followed by three times of washing with PBST buffer. The Dynabeads precipitates were then boiled in sample buffer for 95°C 5 min before loading onto 4-12% SDS-PAGE gels (Novex NP0321Box) for separation and nitrocellulose membrane (Novex LC2000) transfer for western blotting. Proteins were detected with Rabbit anti-NHE6 antibody (Covance 048). 30ug protein were loaded as input to detect NHE6 with above mentioned antibody, NHE9 with Rabbit anti-NHE9 (Covance 050, C-terminal epitope: SPSPSSPTTKLALDQKSSGKC) and α-tubulin with Mouse anti-α-tubulin (Sigma T6074). Western blots were analyzed with Li-CoR Odyssey Imaging System or film development.

### Endosomal acidification analysis in Hap1 ***SLC9A6*** knockout cells

Human Hap1 *SLC9A6* knockout cells and two control cells were loaded with Fluorescein isothiocyanate (FITC)-conjugated transferrin (FITC-Tfn) (Thermo Fisher Scientific T2871) and Alexa Fluor^®^ 546-conjugated transferrin (Alexa Fluor^®^ 546-Tfn) (Thermo Fisher Scientific T23364) as previously described (Xinhan et al., 2011; Ouyang et al., 2013; Ma et al., 2017). In brief, cells were incubated with 66 µg/ml FITC-Tfn and 33 µg/ml Alexa 546-Tfn at 37°C for 10 min, washed twice with PBS, trypsinized, harvested and resuspended in 400ul of Phenol Red-free cell culture medium. Cells were then processed through a cell strainer to generate single-cell populations for flow cytometry/FACS. Standard curve was generated by resuspending cells incubated with FITC-Tfn and Alexa 546-Tfn at 37°C for 30 min in standard buffer solutions containing: 125 mM KCl, 25 mM NaCl, 10 μM Monensin, and 25 mM HEPES (for standards pH 7.0) or 25 MES (for standards pH 6.5, 6.0, 5.5, 5.0) and adjusted to a final pH using 1 N NaOH or 1 N HCl. The mean fluorescence intensity of FITC-Tfn and Alexa 546-Tfn was measured by flow cytometry using BD Influx™ cell sorter (BD Biosciences) and calculated by FlowJo software (Ma et al., 2017). Using the equation generated from standard curve mean intensity, endosomal pH for each experimental sample was calculated.

### RNA Prep and bioanalyzer analysis

Total RNA was isolated from Hap1 CRISPR/Cas9-edited *SLC9A6* knockout cell lines and their control lines using RNeasy Mini Kit (Qiagen 74104) according to the suppliers' instructions. Six replicates each cell line per experiment. rRNA Ratio [28 s/18 s] and RNA integrity number (RIN) of RNA samples were then analyzed by Thermo Scientific™ NanoDrop™ One Microvolume UV-Vis Spectrophotometer and Agilent 2100 Bioanalyzer with Agilent RNA 6000 Nano Kit according to the manufacturer's instruction. All replicas showed rRNA Ratio [28 s/18 s] >=2.0 and RIN is >=9.2 indicating the high quality of RNA extracted (Fig. S7, Table S18). RNA-seq was then performed with poly-(A) enriched RNA.

### RNA-seq data analysis

RNA-seq raw reads for 48 samples were received in Fastq format and analyzed by using two separate pipelines. Illumina universal adapters were detected and removed using ‘Trim Galore’ (version 0.4.0, https://github.com/FelixKrueger/TrimGalore). A representative FastQC report generated from ‘Trim Galore’ to confirm a good quality of reads.

UCSC hg38 was used as a human reference genome for RNA-seq reads alignment and assembly. Both HISAT2-StringTie-DESeq2 pipeline and STAR-HTSeq-DESeq2 pipelines were conducted on High Performance Computing provided by Center for Computation and Visualization (CCV) at Brown University.

### HISAT2 →StringTie→DESeq2 (HISAT2 pipeline)

The pipeline followed a similar process from Pertea's study (Pertea et al., 2016) (Fig. 3). Alignment and the splice junction detection was performed by HISAT2 (version 2.1.0) (Kim et al., 2015). The average coverage was 52 million reads per sample and the average alignment rate was 94.51% per sample (Table S2). Gene and transcript expression levels were estimated by StringTie (version 1.3.3b) (Pertea et al., 2015). The gene expression data were normalized using ‘counts’ function in DESeq2 package. DEGs were identified (*P*.adj<0.01, absolute log2 fold change>1) by using DESeq2 R package (version 1.20.0) (Love et al., 2014).

### STAR→HTSeq→DESeq2 (STAR pipeline)

Alignment and splice junction detection were performed using STAR (version 2.5.3a) (Dobin et al., 2013). Average coverage was 52 million reads per sample, with an average alignment rate of 92% per sample. Gene expression levels were calculated using the ‘htseq-count’ function in HTSeq (version 0.9.1) (Anders et al., 2015). The gene expression data was normalized using ‘counts’ function in DESeq2 package. DEGs were identified (*P*.adj<0.01, absolute log2 fold change>1) by using DESeq2 R package (version 1.20.0) (Love et al., 2014).

MUT4, MUT6 and their paired WT samples were showed in experiment 1 as well as replication experiment. To validate the consistency of DEGs in our model, a Chi-square test was performed on DEGs identified in experiment 1 and the DEGs in replication experiment. In total, 14,951 genes expressed in CS cell model. A contingency table was created. We compared the number of overlapped DEGs from bins of top 50 to top 1000 DEGs in experiment 1 and replication experiment, and the number of overlapped genes if two gene sets were chosen randomly from the total gene list. An extremely high level of concordance of the DEGs was observed between experiment 1 and the replication experiment. The number of overlapped DEGs were significantly enriched in each bin size (*P*<0.001) (Fig. S4C,D).

### WGCNA

WGCNA (Zhang and Horvath, 2005; Langfelder and Horvath, 2008) R package was used to identify the gene co-expression pattern at a system level. The similarity across genes was represented by Pearson's correlation of coefficient. The similarity matrix was then transformed to an adjacency matrix with a power value. Power value was used as a soft threshold, which helped to enlarge the distance between values on a 0-1 scale. The power of 28 was selected.

Unlike unweighted network in which each gene is allowed to collect to one or few other genes and the connection between genes is set to be either 1 or 0 indicating a connection or not, WGCNA allows much more connection across genes. WGCNA sets a small number of highly correlated genes as hub genes if they have a correlation value close to 1 after the power function applied, whereas the correlation value of other non-hub genes will get closer to 0, indicating a weak connection.

Dissimilarity measurement (dissTOM) was then applied on power-transformed adjacency matrix. The dynamic tree cut algorithm was then applied with tree cut at 0.75 and a minimum number restriction of at least 100 genes within each module. Each qualified tree branches formed a module which labelled by color bands as indicated in WGCNA underneath the tree (Fig. 5A).

Gene significance is represented by a Pearson's correlation of the gene expression with sample status, and the corresponding statistical significance measure (*P*-value). The sample status is encoded as binary value for wild type (as 1) or mutant (as −1).

The gene connectivity circle plot was made using the circleplot.R provided by WGCNA. While each module is represented by its first principal component, also called eigengene, the gene connectivity within a module is represented by the Pearson's correlation between the expression of a gene and the eigengene of the module. The higher Pearson's coefficient of correlation, the higher connectivity a gene gets. The circle plot in Fig. 6 shows the connectivity across genes mapped to GO terms of ‘lysosome’ and ‘lysosome membrane’.

GO enrichment analysis of the genes in each module was performed by using DAVID (Huang da et al., 2009a,b) and a false discovery rate (*P*.adj) q value was shown for each enriched GO term.

### Nanostring Custom Panel to validate RNA-seq data

RNA samples were prepared according to Nanostring protocol. In brief, total RNA was extracted from Hap1 NHE6 mutant cell lines and paired control lines. Seven replicates each cell line. 1000 ng of RNA for each sample was prepared for Nanostring.

A pre-designed Nanostring nCounter panel was designed to include 44 genes of interest and six housekeeping genes. Gene expression data were analyzed using nSolver and GeNorm advanced analysis modules provided by Nanostring Technology. Gene expression data was normalized with background subtraction, positive control normalization and the expression of housekeeping genes. The DEGs was identified by comparing the expression of MUT to WT.

## Supplementary Material

10.1242/biolopen.059778_sup1 Supplementary information

Table S1. HAP1 FACS endosomal pH data

Table S2. Alignment summary (Experiment 1 and Replication Experiment)

Table S3. DEGs from Experiment 1 using HISAT2 pipeline

Table S4. DEGs from Experiment 1 using STAR pipeline

Table S5. Nanostring gene list

Table S6. DEGs from Replication Experiment using HISAT2 pipeline

Table S7. DEGs from Replication Experiment using STAR pipeline

Table S8. DEGs from Combined Experiment using HISAT2 pipeline

Table S9. DEGs from Combined Experiment using STAR pipeline

Table S10. Enriched GO terms from common up-regulated DEGs using human refseq as background

Table S11. Enriched GO terms from common down-regulated DEGs using human refseq as background

Table S12. Enriched GO terms from common DEGs using human refseq as background

Table S13. Enriched GO terms from common down-regulated DEGs using detectable genes as background

Table S14. Enriched GO terms from common up-regulated DEGs using detectable genes as background

Table S15. Enriched ingenuity pathways from down-regulated DEGs using IPA

Table S16. Enriched ingenuity pathways from up-regulated DEGs using IPA

Table S17.

Table S18. HAP1 CRISPR-CAS9 SLC9A6 KO lines Bioanalyzer analysis
