# Supplementary material for: Targeting NHE6 gene expression identifies lysosome and neurodevelopmental mechanisms in a haploid in vitro cell model
Source: Biol Open. 2023 Nov 29;12(11):bio059778. doi: 10.1242/bio.059778 (PMC10695175; doi:10.1242/bio.059778)
Supplement: Supplementary information [file biolopen-12-059778-s1.pdf]

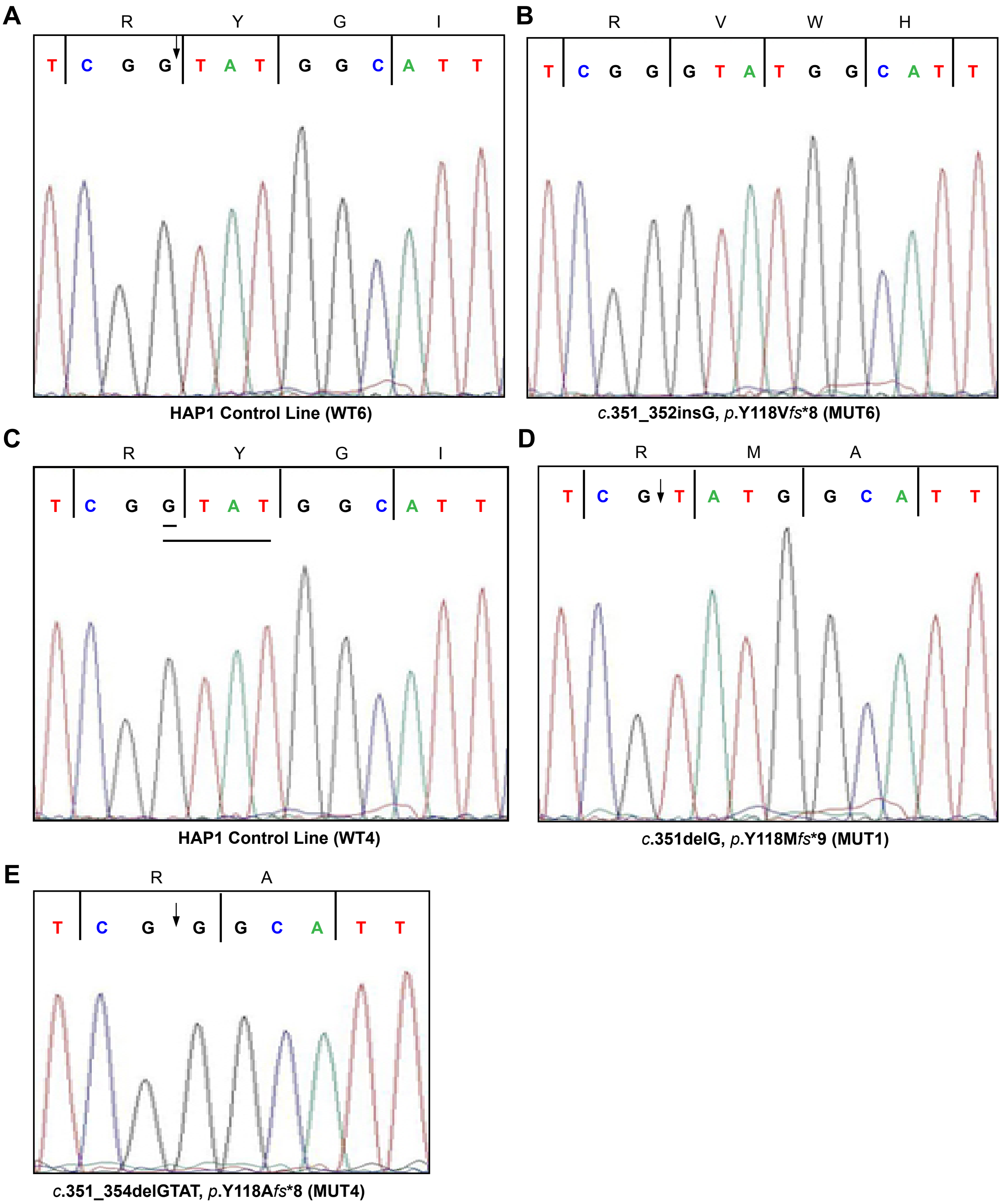


**Fig. S1. Validation of SLC9A6 mutation by Sanger sequencing.** The locations of the mutations are based on NHE6 Transcript NM_001042537. A, B. Sanger sequencing is shown of HAP1 control line (A, WT6) and 1bp insertion mutation line (B, MUT6). Asterisk indicates the insertion of nucleotide G, reading frame was changed with translational amino acid change at Y118 and early stop codon generated. C, D, E. Sanger sequencing is shown of HAP1 control line (C, WT4) and 1bp deletion (D, MUT1) and 4bp deletion mutation line (E, MUT4). Arrows indicate the deletion position, similarly, reading frame was changed with translational amino acid change at Y118 and early stop codon generated.

Biology Open • Supplementary information

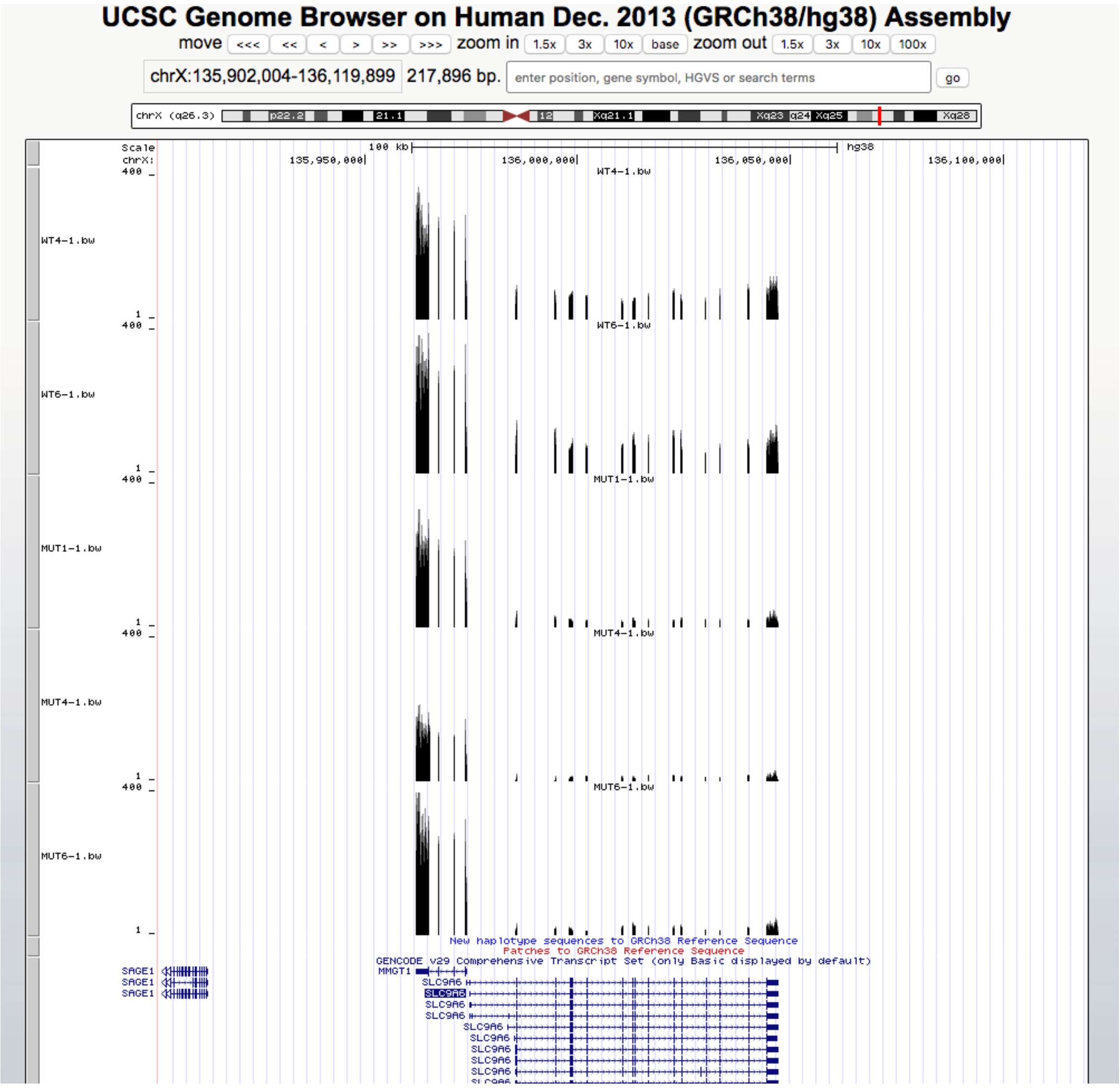


**Fig. S2. UCSC genome browser view of RNA-seq samples on SLC9A6 location.** X- and Y- axes represent the genomic position on UCSC/hg38 and sequencing read depth. Two wild types (WT4 and WT6) showed a higher read depth than mutants (MUT1, MUT4 and MUT6).

Biology Open • Supplementary information

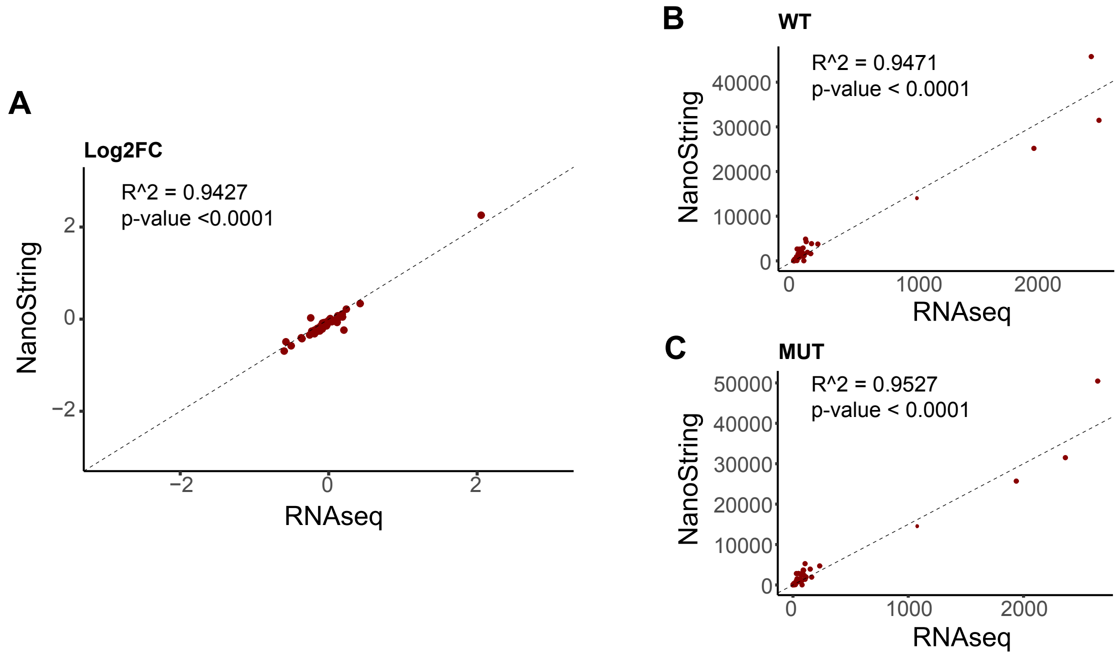


**Fig. S3. Validation of RNA-sequencing gene expression results by comparison with Nanostring data sets.**

A. Log2 fold change of gene expression from RNA-seq and Nanostring technology. $R^2$ and p were calculated from a linear model. The line y=x is shown for a positive correlation reference. B. Gene expression data of wild-type samples generated from RNA-seq and Nanostring technology. $R^2$and p value were calculated from a linear model. y=0.1x is shown for a positive correlation reference.

C. Gene expression data of mutant samples generated from RNA-seq and Nanostring technology. $R^2$and p value were calculated from a linear model. The line y=0.1x is shown for a positive correlation reference.

Biology Open • Supplementary information

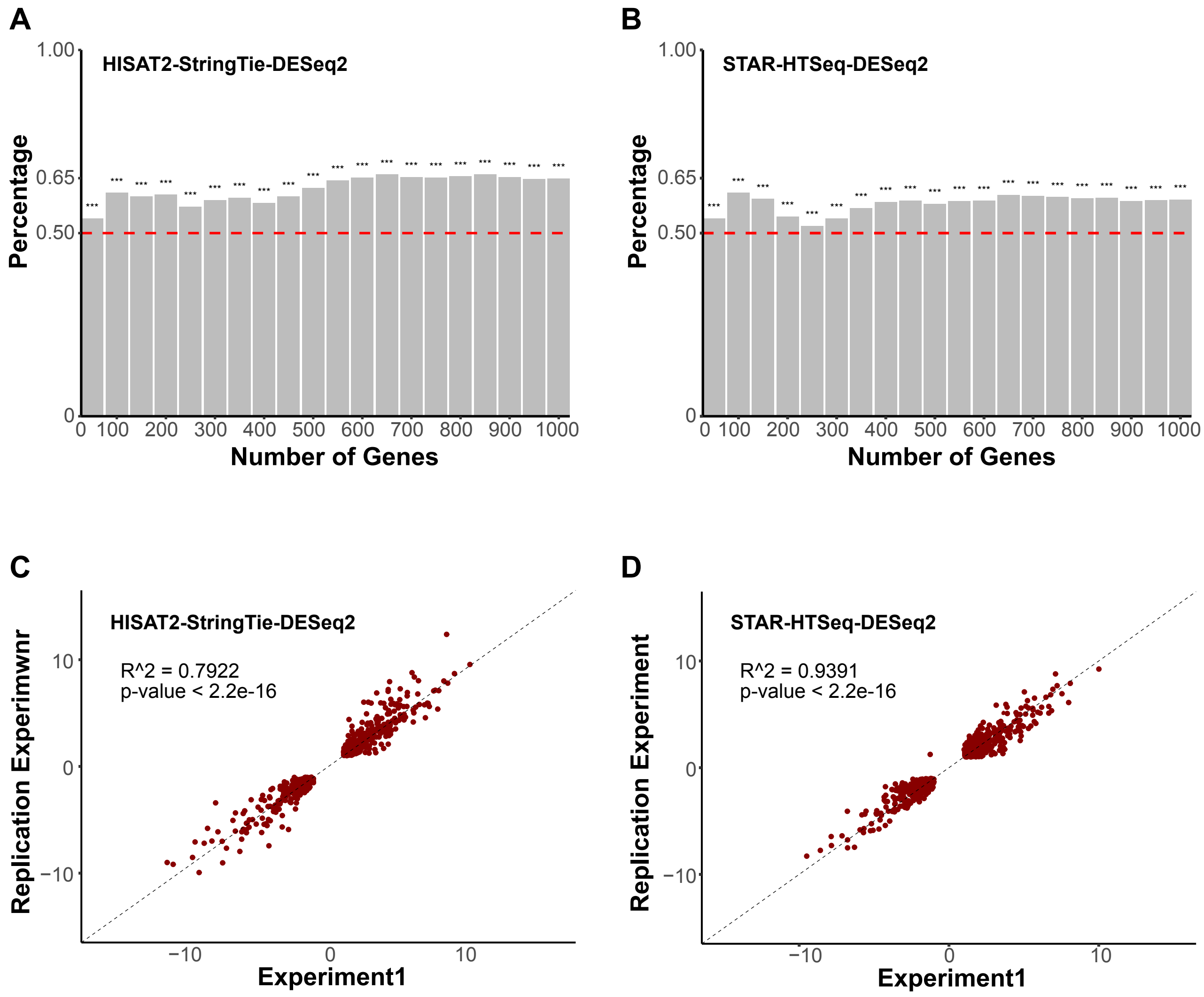


**Fig. S4. Comparison of DEGs between experiments and between pipelines.** DEGs are generated by (A) Experiment 1 and Replication Experiment using HISAT2-StringTie-DESeq2 pipeline, (B) Experiment 1 and Replication Experiment using STAR-HTSeq-DESeq2 pipeline. Comparison of Log2 fold change of DEGs generated by (C) Experiment 1 and Replication experiments using HISAT2-StringTie-DEseq2 pipeline and (D) Experiment 1 and Replication experiments using STAR-HTseq-Deseq2 pipeline. DEGs are defined as p < 0.01, log2 Fold Change > 1, and are sorted by their P.ADJ value.

Biology Open • Supplementary information

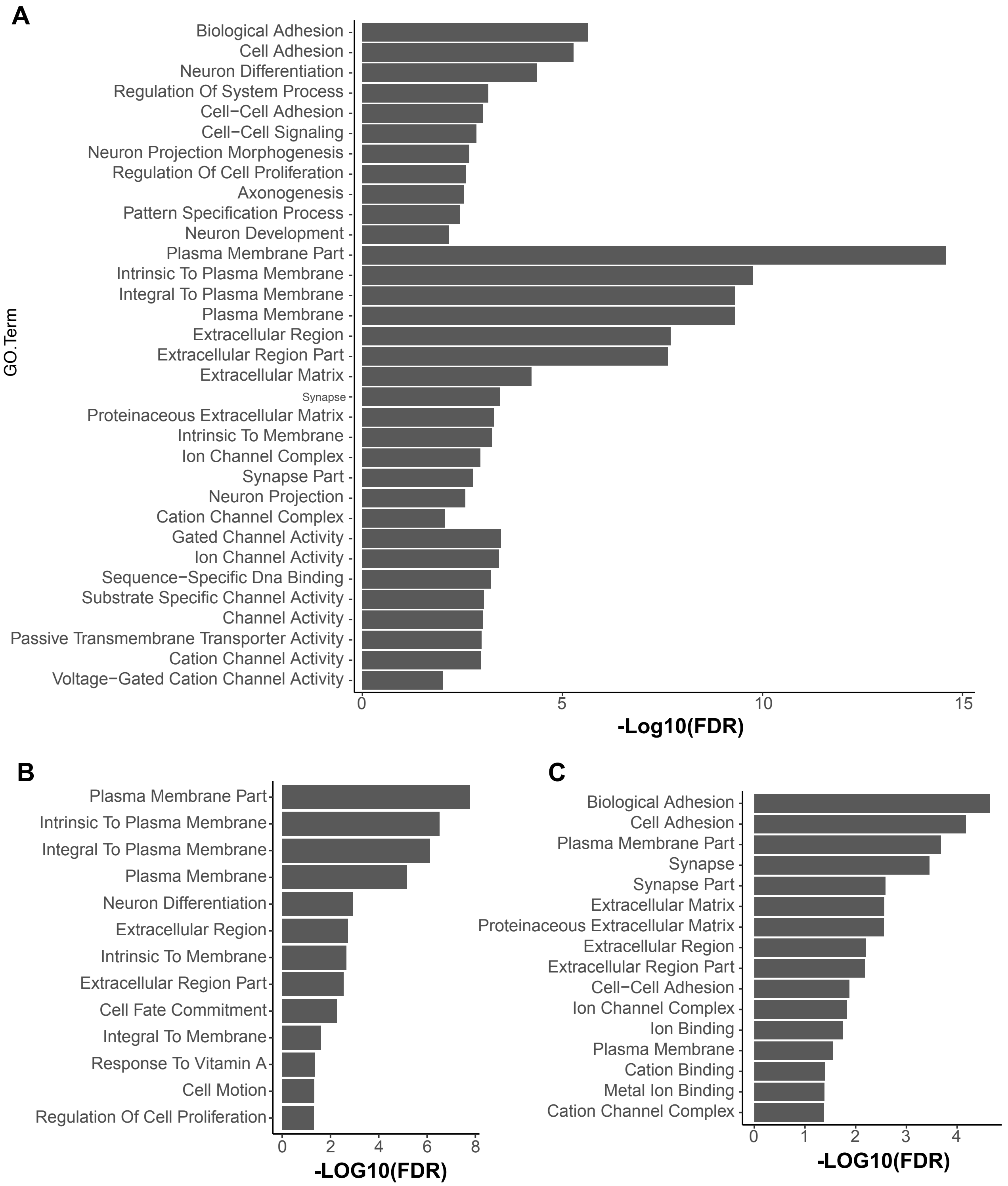


Biology Open • Supplementary information

**Fig. S5. Bar chart of enriched gene ontology terms for (a) common, (b) common upregulated and (c) common down-regulated genes (P.ADJ < 0.05).** 1056 DEGs commonly identified by HISAT2-StringTie-DESeq2 and STAR-HTSeq-DESeq2 pipelines were analyzed in DAVID. Among these genes, 586 up-regulated genes and 470 down-regulated genes. (d) Pathway analysis using ingenuity pathway analysis (IPA®) of 1056 DEGs detected by both HISAT2-StringTie-DESeq2 and STAR-HTSeq-DESeq2 pipelines. A Bar chart showing significantly overrepresented pathways. Up- and down-regulated genes were shown in red and green, respectively.

Biology Open • Supplementary information

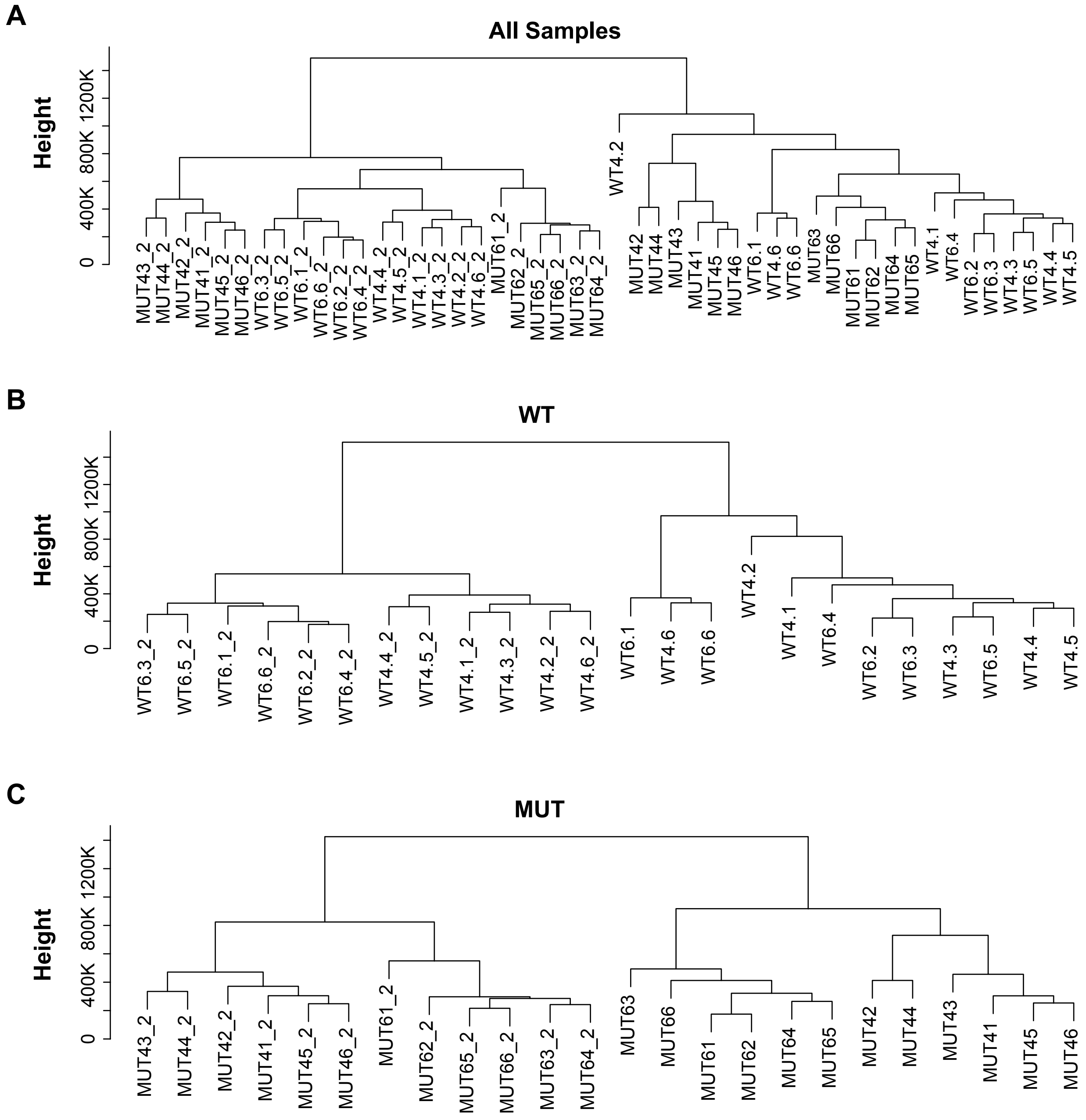


**Fig. S6. Hierarchical clustering dendrogram on (A) all 48 samples, (B) wild-type samples, (C) mutant samples.**

Biology Open • Supplementary information

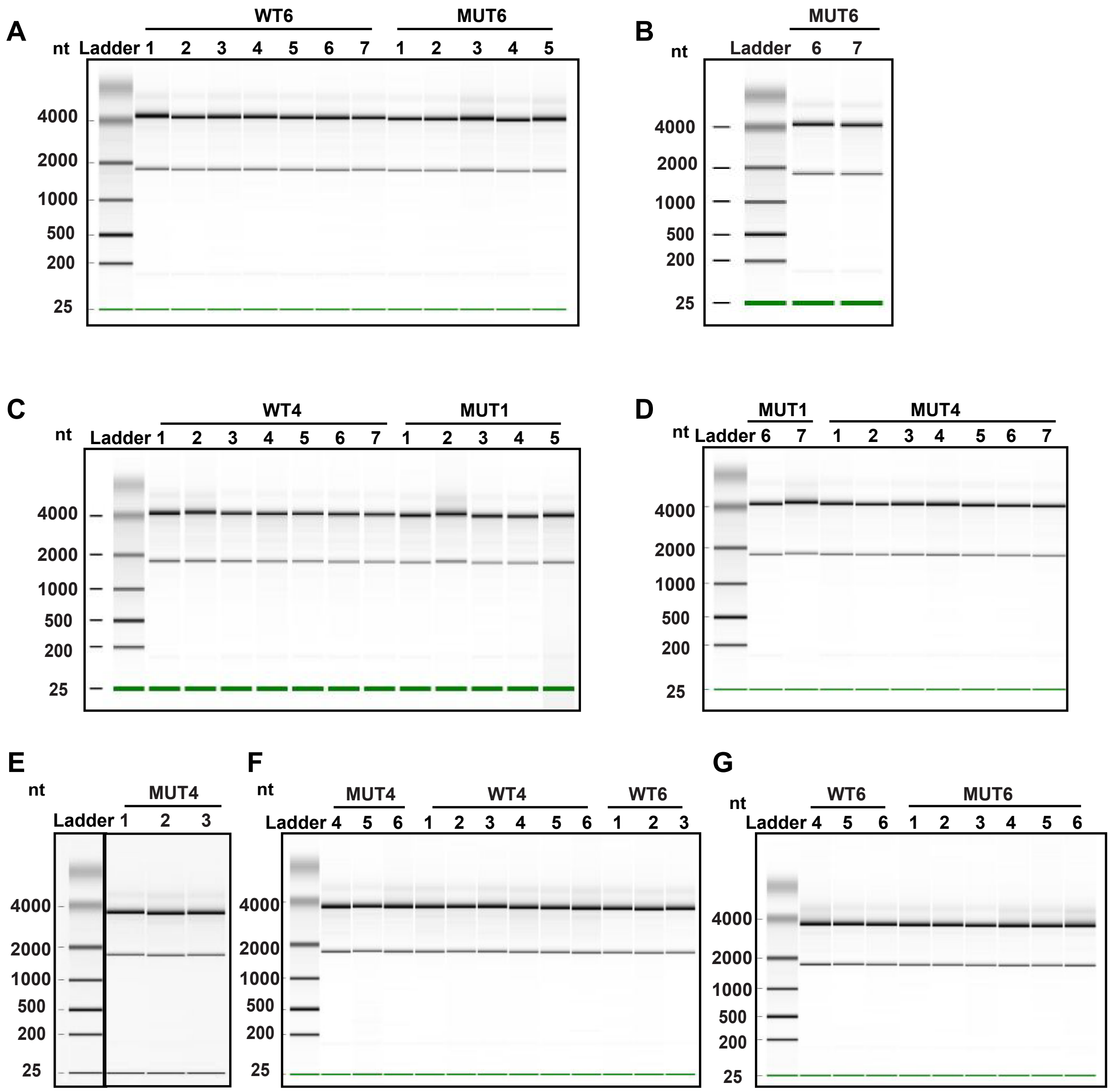


**Fig. S7. Agilent bioanalyzer gel-like image of total RNA.** The images show a total RNA gel like-image produced by the bioanalyzer. Size makers was shown on the first lane for each image. The 28s and 18s distinctive ribosomal RNA bands are observed for all samples.
A, B. Seven RNA replicas of WT6 and MUT6 for Experiment 1 RNA-seq;
C, D. Seven RNA replicas of WT4, MUT1 and MUT4 for Experiment 1 RNA-seq.
E, F, G. Six RNA replicas of MUT4, WT4, WT6 and MUT6 for Replication Experiment RNA-seq.

Biology Open • Supplementary information

**Table S1. HAP1 FACS endosomal pH data**

Click here to download Table S1

**Table S2.** Alignment summary (Experiment 1 and Replication Experiment)

Click here to download Table S2

**Table S3.** DEGs from Experiment 1 using HISAT2 pipeline

Click here to download Table S3

**Table S4.** DEGs from Experiment 1 using STAR pipeline

Click here to download Table S4

**Table S5.** Nanostring gene list

Click here to download Table S5

**Table S6**. DEGs from Replication Experiment using HISAT2 pipeline

Click here to download Table S6

**Table S7.** DEGs from Replication Experiment using STAR pipeline

Click here to download Table S7

**Table S8.** DEGs from Combined Experiment using HISAT2 pipeline

Click here to download Table S8

**Table S9.** DEGs from Combined Experiment using STAR pipeline

Click here to download Table S9

**Table S10.** Enriched GO terms from common up-regulated DEGs using human refseq as background

Click here to download Table S10

**Table S11.** Enriched GO terms from common down-regulated DEGs using human refseq as background

Click here to download Table S11

**Table S12.** Enriched GO terms from common DEGs using human refseq as background

Click here to download Table S12

**Table S13.** Enriched GO terms from common down-regulated DEGs using detectable genes as background

Click here to download Table S13

**Table S14.** Enriched GO terms from common up-regulated DEGs using detectable genes as background

Click here to download Table S14

**Table S15.** Enriched ingenuity pathways from down-regulated DEGs using IPA

Click here to download Table S15

**Table S16.** Enriched ingenuity pathways from up-regulated DEGs using IPA

Click here to download Table S16

**Table S17.**

Click here to download Table S17

**Table S18.** HAP1 CRISPR-CAS9 SLC9A6 KO lines Bioanalyzer analysis

Click here to download Table S18
